# Analysis of sea use landscape pattern based on GIS: a case study in Huludao, China

**DOI:** 10.1186/s40064-016-3038-z

**Published:** 2016-09-15

**Authors:** Anning Suo, Chen Wang, Minghui Zhang

**Affiliations:** 1 National Marine Environmental Monitoring Center, Dalian, 116023 People’s Republic of China; 2 Key Laboratory of Sea Field Management Technology, SOA, Dalian, 116023 People’s Republic of China; 3 Satellite Environment Center, Ministry of Environmental Protection, Beijing, 100094 China; 4 College of Civil Engineering, Dalian Ocean University, Dalian, 116023 China

**Keywords:** Sea use, Marine, Landscape pattern, Analysis, GIS

## Abstract

This study aims to analyse sea use landscape patterns on a regional scale based on methods of landscape ecology integrated with sea use spatial characteristics. Several landscape-level analysis indices, such as the dominance index, complex index, intensivity index, diversity index and sea congruency index, were established using Geographic Information System (GIS) and applied in Huludao, China. The results indicated that sea use landscape analysis indices, which were created based on the characteristics of sea use spatial patterns using GIS, are suitable to quantitatively describe the landscape patterns of sea use. They are operable tools for the landscape analysis of sea use. The sea use landscape in Huludao was dominated by fishing use with a landscape dominance index of 0.724. The sea use landscape is a complex mosaic with high diversity and plenty of fishing areas, as shown by the landscape complex index of 27.21 and the landscape diversity index of 1.25. Most sea use patches correspond to the marine functional zonation plan and the sea use congruency index is 0.89 in the fishing zone and 0.92 in the transportation zone.

## Background

Sea use refers to human activities (e.g., fishing, navigation, constructions and aquaculture) in the exploitation of marine resources (Miao 2004; Zhang 2004; Luan and Li 2008; Wang 2002). Following the rapid development of the marine economy, large areas of natural marine surface have been explored all over the world, resulting in serious environmental and resource losses, such as habitat degradation, biodiversity loss, as well as pollution and eutrophication (Yun 2005; Yu and Qi 2006; Wang and Zhang 2007). Within the context of these losses, wide attention has recently been drawn to the management of the multi-purpose use of sea space in many countries (Xu et al. 2006; Ma et al. 2008; Sun et al. 2010, 2012). The development of monitoring, impact assessment and research methods for sea use is particularly warranted, considering the important and valuable ecosystem services destroyed by unsustainable sea use (O’Neill et al. 1988).

Landscape ecology focuses on interactions between spatial patterns and ecological processes in landscape scale, and has been increasingly applied in assessing and monitoring of landscape health (Bertollo 2001; Turner et al. 2001; Arsanjani et al. 2016). Landscape pattern indices measure landscape spatial attributes, such as spatial composition and configuration, which could serve as effective indicators in understanding landscape changes, identifying pressure points, improving utility demand, adopting relative polices and constructing effective plans for sustainable development (Waddell 2002; Barredo and Demicheli 2003; Vaz et al. 2012; Vaz 2014). The analysis of landscape pattern based on landscape indices has been widely applied to terrestrial ecosystems, such as forest, urban, agriculture and wetland (Saura et al. 2011; Tv et al. 2012; Wu 2013; Plexida et al. 2014), and is proven to be a powerful tool to investigate spatial dynamics of land use in a large scale (Wu 2013; Arsanjani et al. 2016). However, the application of landscape ecology theories and methods in evaluating regional sea use on a macro-scale is a new field in landscape ecology studies, which is rather limited to our knowledge (Vaz et al. 2013; Vaz 2014, 2016).

Similar to the spatial pattern of land use, the sea use pattern is a spatial mosaic of sea use patches on the matrix of the natural marine surface (Suo et al. 2009). However, the sea use landscape is more complex compared with the land use landscape (Suo et al. 2014). Some sea use patches have obvious boundary lines, as with land use, such as salt ponds, fishing ponds and land reclamation. Some sea use patches are space partitioned for the exploitation of marine resources without clear boundary lines, such as sea-routes and anchorages. Some other sea use patches (e.g., sea-water baths and artificial reefs) have markers for boundary line; these are, however, too small to be detectable using remote sensing. Due to the spatial complexity of the sea use pattern, it is difficult to analyse sea use landscape via traditional methods of landscape ecology.

Geography Information System (GIS) has enormous advantages in processing and analysing sea use spatial data. For example, it can transfer and standardise the spatial data with different scales and coordinate systems and can also link the non-spatial data such as attributed data. Thus, GIS is expected to be useful in the analysis of sea use with its powerful spatially explicit properties (Jin et al. 2008). The establishment of special landscape pattern index for sea use is an important task for the management of sea space and the study of marine macro ecology. Aiming to analyse the landscape pattern of sea use in the regional scale, this paper used GIS as a basic tool to establish five indices and applied them to the landscape analysis of sea use in Huludao, China.

## Study area

**Fig. 1:**
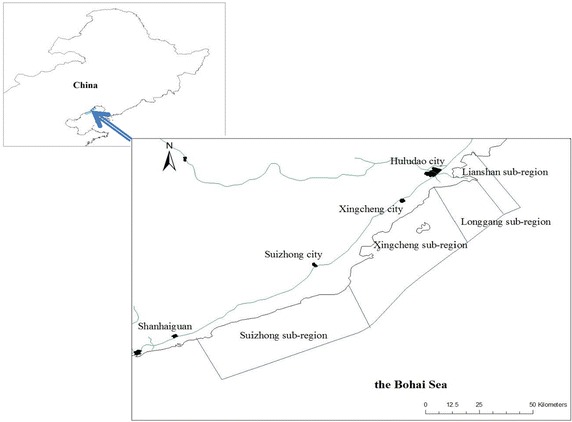
Location of study area

Huludao is located in the southwest of Liaoning province in China, with latitude 39°52′–42°24′N and longitude 119°50′–121°05′E (Fig. 1). It faces the Liaodong Bay of the Bohai Sea in the south, with a total sea area of 357,748.06 hm^2^, serving as an important transport corridor between Northeast China and Northern China. It includes four coastal sub-regions, which are Suizhong, Xingcheng, Longgang and Lianshan. The coastline in the Huludao region is 258 km long, most of which is sandy coast. The sea field in Huludao is suitable for various types of sea use in terms of its 60 % shallow sea areas (above 10 m depth line). In total, 28,726.36 hm^2^ of the sea field landscape in Huludao are covered with different types of sea use including fishing pond, salt pond, reclamation of sea area for building, sea sand exploitation, port and sea-route, as well as sea-water baths and beach.

## Sea use landscape pattern indices

Sea use landscape pattern is similar to landscape pattern in land use, where the marine surface is a landscape matrix—like grassland or a forest on land—and sea use patches are landscape mosaics. The marine functional zonation plan is a marine spatial planning made for sea use management. Similar with land use planning, the marine functional zonation plan requires use of the sea area in accordance with the planned spatial function divisions. In this paper, several indices were selected to objectively evaluate the spatial characteristic of the landscape pattern of sea use. The index of sea use landscape dominance was selected to describe the dominant type of sea use in a region and its dominant status. The index of sea use landscape complex was selected to describe the shape complexity of sea use patches in a region. The index of sea use landscape diversity was selected to describe the diversity of sea use types and area ratio in a region. The index of sea use landscape intensity was selected to describe the scale of the actual sea use in the marine functional zonation. The index of sea use landscape congruency was selected to describe the correspondence of the sea use type and area to the marine functional zonation plan. The indices are calculated as follows:

### (1) Sea use landscape dominance

Sea use landscape dominance is the most dominant of a certain sea use type in a regional sea field landscape. It can be described by the sea use landscape dominant index as follows:

1 $$ D = \sum\limits_{i = 1}^{m} {N_{i}^{{}} } $$

2 $$ N_{i}^{{}} = (P_{i} + M_{i} )/2 $$

where *D* is the sea use landscape dominant index, *N*_*i*_ is the importance value of sea use type *i*, *P*_*i*_ is the area ratio of sea use type *i* to total areas of the sea field landscape, and *M*_*i*_ is the patch number ratio of sea use type *i* to total patch number of the sea field landscape.

### (2) Sea use landscape complex

Sea use landscape complex is the complexity of the sea use landscape pattern. It can be described by the patch shape index of sea use as follows:

3 $$ HSI = \frac{0.25E}{\sqrt A } $$

where *HSI* is the patch shape index of sea use, *E* is the total length of the boundary lines of the sea use patches, and *A* is the total areas of sea use patches.

### (3) Sea use landscape diversity

Sea use landscape diversity is the diversity of sea use types and the composition of sea use patches. It can be described by the sea use landscape diversity index as follows:

4 $$ HYDI = - \sum\limits_{i = 1}^{m} {[P_{i} \ln (P_{i} )} ] $$

where *HYDI* is the sea use landscape diversity index, and *HYDI* ≥ 0. *P*_*i*_ is the area ratio of sea use type *i* to the total areas of the sea field landscape, and *m* is the number of sea use types. When there is only one sea use type in the landscape, *HYDI* = 0. When the number of sea use types increases or when the area ratio of all sea use types is similar, the relative *HYDI* increases.

### (4) Sea use landscape intensity

Sea use landscape intensity is the area ratio of the actual sea use relative to the marine functional zonation plan. The sea use can be divided into seabed use, sea body use and sea surface use, considering the three dimensions of the marine space (Liu and Su 2005). Sea use landscape intensity can be described by the sea use landscape intensivity index as follows:

5 $$ SUS = \frac{{\sum\nolimits_{i = 1}^{n} {a_{i} } }}{{A_{u} }} \times 100\,\% $$

where *SUS* is the sea use landscape intensivity index, and *SUS* ≥ 1. *A*_*u*_ is the total area of marine function zone *u*. *a*_*i*_ is actual sea use areas of type *i*.

### (5) Sea use landscape congruency

Sea use landscape congruency is the correspondence of a certain sea use type to its marine functional zonation plan. It can be described by the sea use congruency index as follows:

6 $$ FH = 1 - \sum\limits_{i = 1}^{n} {wa_{i} } $$

where *FH* is the sea use congruency index, 0 ≤ *FH* ≤ 1.0, and *w* is the congruency judgment weight. If the sea use type corresponds to the marine functional zonation plan, *w* = 0. If the sea use type does not correspond to the marine functional zonation plan, *w* = 1.0. *a*_*i*_ is area ratio of sea use type *i* in the marine functional zonation plan. The bigger the landscape congruency index is, the more the sea use types correspond to the marine functional zonation plan. When the patches of all the sea use types correspond to the marine functional zonation plan, *FH* = 1.0; otherwise, *FH* = 0. The correspondence of sea use types to different marine functional zones is shown in Table 1.

**Table 1:** Correspondence of sea use types to marine functional zones

Function zones	Sea use types							
	Fishing	Transportation	Industry and town	Tour	Submarine cables and pipelines	Pollution	Milning	Sea salt
Fishing zone	√	X	X	√	X	X	X	X
Transportation zone	X	√	X	X	X	√	X	X
Industry and town zone	√	X	√	X	X	√	√	X
Tour functional zone	√	X	X	√	√	X	X	√
Mining zone	√	X	√	X	X	√	√	X
Conservation zone	√	X	X	√	X	X	X	√
Reservation zone	√	X	X	X	X	X	X	√
Special use zone	X	X	X	X	X	X	X	√

√ represents sea use type is permitted in the marine functional zone, X represents sea use type is not permitted in the marine functional zone

## Data and process

**Fig. 2:**
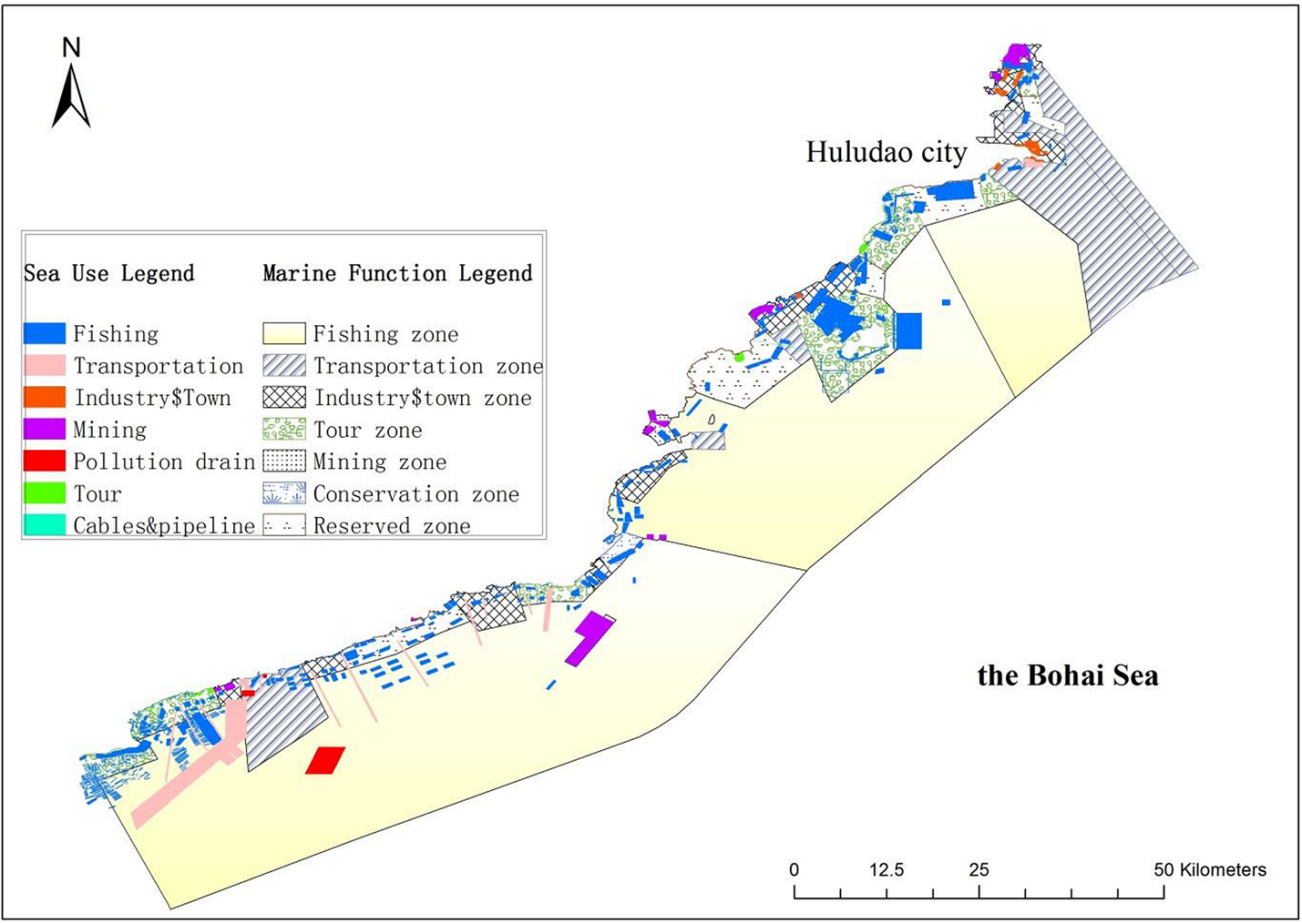
Sea use patches overlapped on marine function correspondence map in Huludao

The analyses in this paper were based on data of sea use ownership, the marine functional zonation plan, coastline and the marine administrative boundary for the sea field of Huludao. The sea use ownership data was mapped by field surveys of vertexes of ownership patches with GPS. The marine functional zonation plan, coastline and marine administrative boundary were provided by the Huludao government. The sea use ownership data was spatially overlapped with the maps of the marine functional zonation plan, coastline and the marine administrative boundary (Fig. 2). The area and perimeter of each sea use patch is calculated by XTools Pro, which is integrated in ArcGIS Desktop 10.0. The sea use landscape dominant index is calculated by accumulating an importance value of each sea use type, which is the average of the statistical areas ratio and statistical patch number ratio for each sea use type. The patch shape index is calculated by total area of each sea use type and total perimeter of each sea use type. The sea use landscape diversity index is calculated by accumulating the area ratio of sea use type *i* to total sea use in a region, as in formula (4).

**Fig. 3:**
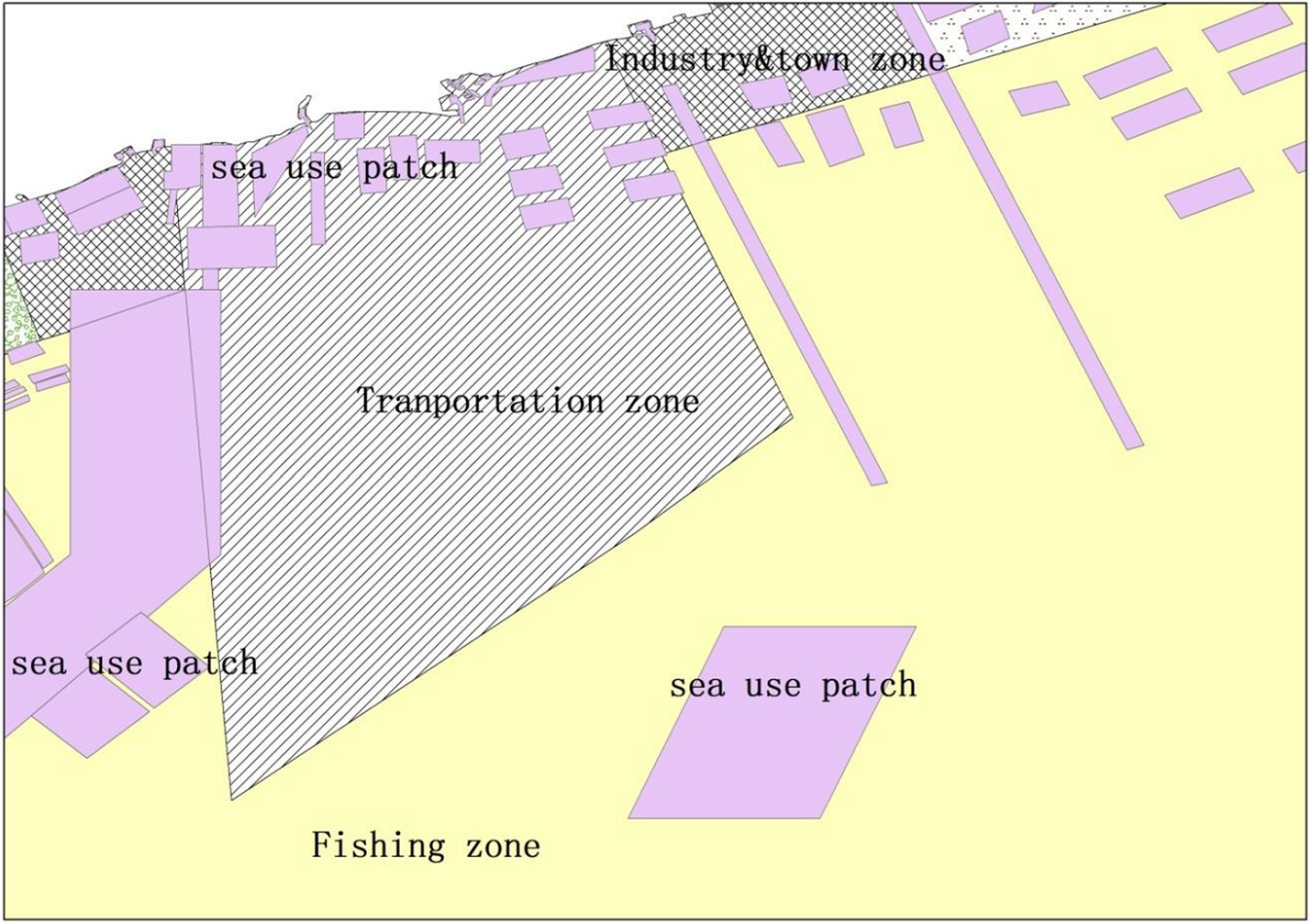
Sea use patches overlapped on marine functional zones

**Fig. 4:**
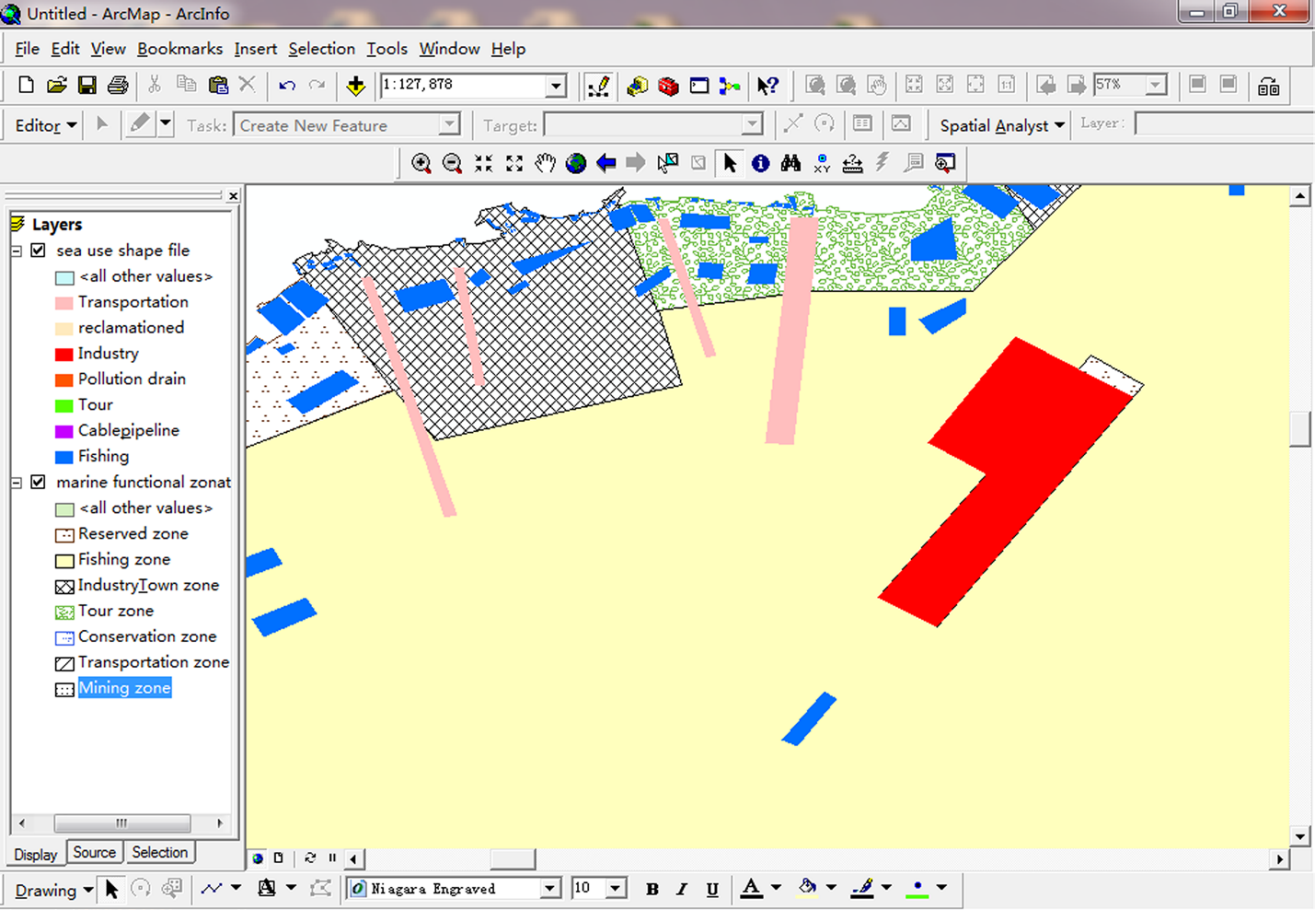
Screen shot of sea use patches corresponding to marine functional zones

The sea use landscape intensivity and sea use congruency indices are calculated using the marine zonation map. The sea use landscape intensivity is assessed, using formula (5), by calculating the area of overlap between various types of zonation in the marine zonation plan and various sea use patches (for an example see Fig. 3). Sea use congruency is assessed by the sea use patch type and its location within the various marine zones. The area of sea use patches which correspond to each marine functional zone are added and used to calculate the sea use congruency index using formula (6) (see Fig. 4).

## Results and analyses

Table 2 shows the landscape dominant index of different sea use types in the sea field of Huludao. Fishing area is the dominant type with a landscape dominant index of 0.724. Being the most common sea use type, it occupies 58.25 % total area and 85.66 % of the sea use patch number. This illustrates that the fishing patches are numerous, but small. Transportation is the next most dominant type, with a landscape dominant index of 0.145. The area ratio for transportation is 23.89 %, but the patch number ratio is only 5.09 %, indicating less patches of transportation with, but with a large area. The landscape dominant index for other sea use types are all below 0.10, which reveals the small proportion occupied by these types in the total area and the total sea use patch number. The area ratio for mining, sea salt, pollution, industry and town, tour, submarine cables and pipelines are 5.59, 4.36, 4.12, 2.11, 1.04 and 0.65 %, respectively.

**Table 2:** Landscape dominant index and patch shape index of different sea use types in Huludao

Sea use types	Areas ratio (%)	Patch number ratio (%)	Importance value	Dominant index	Patch shape index
Fishing	58.25	86.56	72.41 %	0.724	26.29
Mining	5.59	0.29	2.94 %	0.029	1.63
Industry and town	2.11	1.63	1.87 %	0.019	4.61
Submarine cables and pipelines	0.65	0.29	0.23 %	0.002	8.41
Pollution	4.12	0.29	2.21 %	0.022	1.44
Tour	1.04	1.25	1.15 %	0.012	3.99
Sea salt	4.36	2.02	3.19 %	0.032	4.11
Transportation	23.89	5.09	14.49	0.145	8.26

The patch shape index of the whole sea use landscape (27.21) is similar to the shape index of fishing patches (26.29)—a high value due to their complex shape. The patch shape of other sea use types is relatively simple (Table 2). The patch shape index of submarine cables and pipelines is 8.41 for the banding distribution of patches. The patch shape index for transportation is 8.26 for the banding distribution of sea-route. The patch shape index of industry and town, sea salt, tour, pollution and mining are 4.61, 4.11, 3.99, 1.44 and 1.63, respectively.

**Fig. 5:**
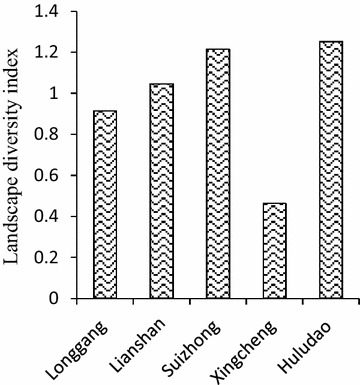
Landscape diversity index in Huludao

**Fig. 6:**
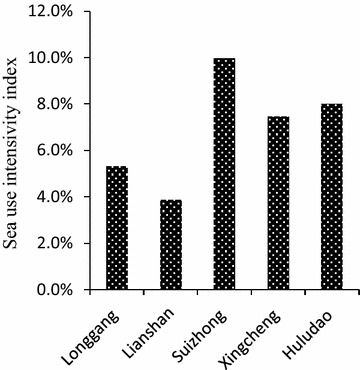
Sea use intensivity index in Huludao

The sea use landscape diversity index is 1.251 in Huludao as a whole and different in the four sub-regions (Fig. 5). The highest value of 1.215 is obtained in Suizhong, while the smallest is in Xingcheng, which is only 0.463. The sea use landscape diversity index is similar in Longgang (0.913) and Lianshan (1.045). Most of the sea use is concentrated on the marine surface in Huludao and there are a few areas with bi-layered or three-layered sea use structures. The total sea use area is 28,726.36 hm^2^, which accounts for 8.02 % of the sea field in Huludao. The sea use intensivity index is the highest in Suizhong (9.97 %), followed by Xingcheng with a value of 7.46 %. It is only 5.31 % in Longgang and 3.87 % in Lianshan (Fig. 6).

**Fig. 7:**
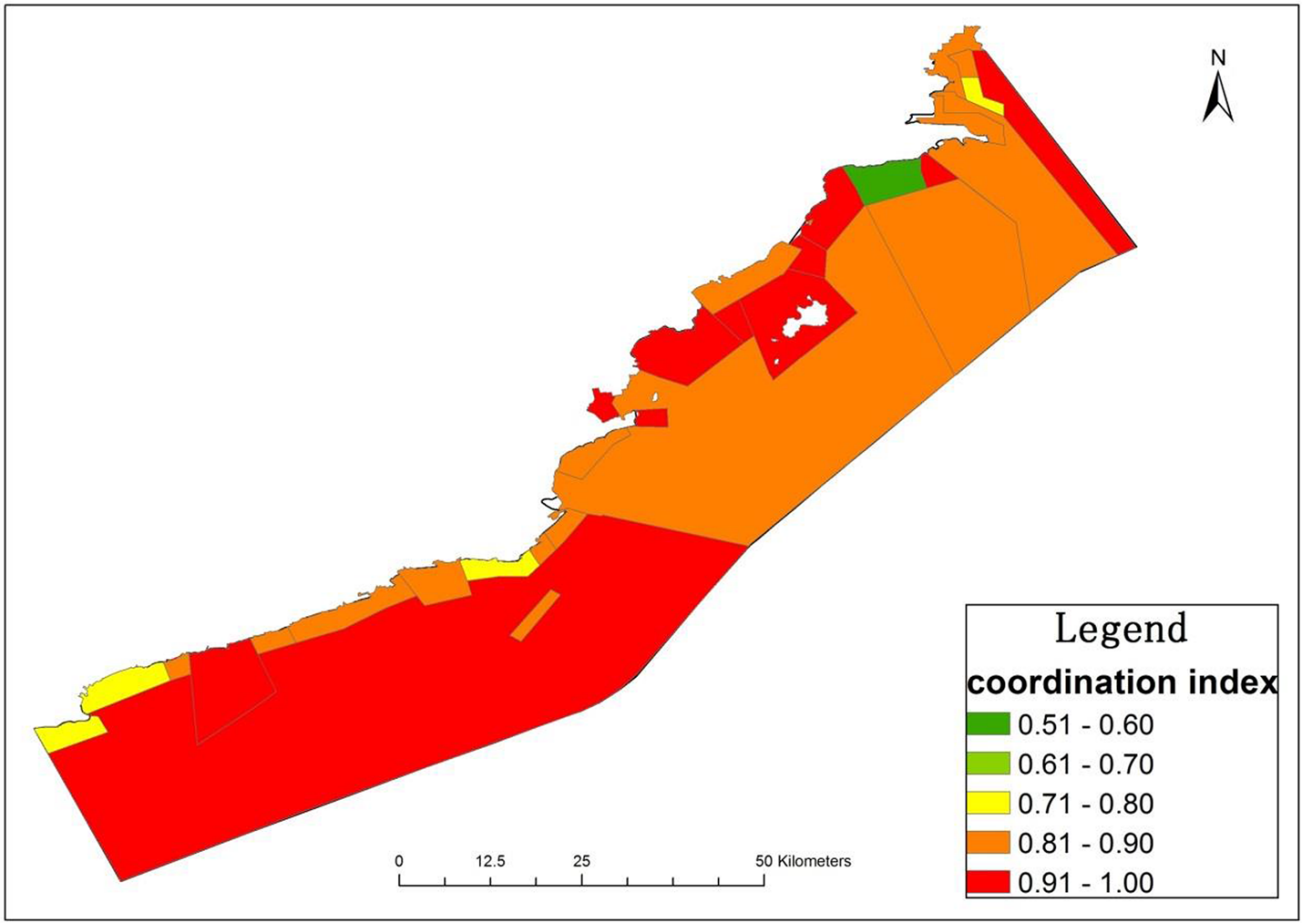
Sea use congruency index in Huludao

The marine functional zonation plan is the basic spatial control rule for sea use management. However, some sea use activities do not correspond to the marine functional zonation plan in Huludao because of historical continuity and illegal operation. Figure 7 shows the sea use congruency index of the main marine functional zonation plan in Huludao. The fishing zone is the largest zone patch, according to the marine functional zonation plan, which accounts for 76.24 % of the total area of the sea field. The sea use congruency index of the fishing zone is 0.89 for the patches of diverse sea use types mosaicking in the fishing zone. The transportation zone is the second biggest zone with area 37,435.81 hm^2^ and the highest congruency index of 0.92. The second biggest congruency index is observed in the mining zone with a value of 0.91, whilst being the smallest in the conservation zone, with a value of 0.80. The congruency index of sea use is different in the four sub-regions for different types of the marine functional zonation plan and various patches of spatial sea use. Suizhong contains five marine functional zonation plan types, with the biggest sea use congruency index being the fishing zone (0.95), followed by the transportation zone (0.93), the industry and town zone (0.89), conservation zone (0.80) and tour zone (0.72). The sea use congruency index is smaller in Xingcheng, which reaches around 0.90 in most zones and falls to 0.84 in the fishing zone. The marine functional zonation plan is simpler in Longgang and Lianshan with only four, or three, types of functional zonation, the congruency index of which is below 0.90 in most functional zonation.

## Discussion

The landscape pattern index is a quintessential method to quantitatively describe the landscape pattern, which has been widely applied in land use landscape, vegetation landscape, wetland landscape and so forth (Saura et al. 2011; Plexida et al. 2014). However, there is rarely any research focusing on the sea use landscape, the reason for which is twofold. On the one hand, the development and utilisation of marine space is relatively low, with few sea use types and simple spatial patterns in many countries that makes the quantitative evaluation of sea use landscape less developed. On the other hand, the sea use landscape is largely different from the terrestrial landscape of land use and vegetation, which makes it difficult to obtain sea use landscape data using effective methods for terrestrial landscape such as satellites.

Recently, the increased complexity in sea use landscape makes the quantitative description and management of the sea use in space a main technical problem and challenge for the marine management in China, especially following the increased intensity of marine development, as well as the increased size and type of sea use. As a result of the large difference between the sea use landscape and the terrestrial landscape of land use, vegetation and wetlands, it is difficult to accurately describe the landscape pattern of sea use using a traditional landscape pattern index (Tv et al. 2012). To face the challenge, this study developed five landscape pattern indices especially for sea use, which are landscape dominant index, patch shape index, landscape diversity index, landscape intensivity index, and the congruency index of sea use. To test the efficiency of these indices we successfully applied them in evaluating the sea use landscape in Huludao. To our knowledge—based on the extant literature—this research is the first to study the sea use landscape using landscape pattern index. Considering the potential limitation of these indices in describing characteristics of sea use landscape patterns, further improvement of the quantitative analysis method of sea use landscape is needed in the future.

## Conclusion

Although the potential application of GIS in sea use analysis and management has been recognised by some researchers, there are few analysis reports of sea use from the viewpoint of landscape. The management of sea use in China requires a system of analysis methods from landscape scale to enhance the control on the marine exploitation activities. This paper established five analysis indices of sea use, based on GIS, and applied them in the Huludao region. The results showed that the sea use analysis indices, based on GIS, are suitable and effective for the assessment and evaluation of sea use on a landscape scale. The sea use landscape is dominated by fishing in the Huludao, with a dominant index of 0.724. The numerous, but small, fishing patches scatter in all types of sea functional zonation. In general, the sea use landscape is complex, with patch shape index as high as 27.21 and a diversity index of 1.25. Most sea use patches are in accordance with the marine functional zonation plan, indicated by the congruency index of 0.89 in the fishing zone and 0.92 in the transportation zone.

The coastal zone is becoming more globalised and socioeconomic development is increasing, following the development of global economy. Similar to the landscaping patterns of terrestrial land use, the footprints of human activities in the coastal waters form the landscaping pattern of sea use, such as the Palm islands in Dubai, the Narita International Airport in Japan and offshore aquaculture with floating rafts and cages. Such sea use landscaping could become important regions for coastal living, tourism, transportation and the fishing industry with proper planning and management. However, unreasonable sea use would lead to a number of environmental problems, such as coastal wetland destruction, coastal erosion or deposition, marine environmental pollution and biodiversity degradation. Therefore, it becomes the primary mission for coastal managers to oversee this complex sea use issue with insight. The sea use landscape index studied in this paper is an important quantitative method for the spatial pattern of sea use, which could serve as a useful tool for the evaluation and planning of the sea use on a spatial scale.
